# SENP1 promotes triple-negative breast cancer invasion and metastasis via enhancing CSN5 transcription mediated by GATA1 deSUMOylation

**DOI:** 10.7150/ijbs.60594

**Published:** 2022-03-06

**Authors:** Yongchang Gao, Rongrong Wang, Jianjing Liu, Ke Zhao, Xiaolong Qian, Xianghui He, Hong Liu

**Affiliations:** 1 Department of General Surgery, Tianjin Medical University General Hospital, Tianjin, China.; 2 Department of Obstetrics and Gynecology. Tianjin Medical University General Hospital, Tianjin, China.; 3 Department of Nuclear Medicine and Molecular Imaging, Tianjin Medical University Cancer Institute and Hospital, National Clinical Research Center for Cancer, Tianjin, China.; 4 Department of Breast Cancer Pathology, Tianjin Medical University Cancer Institute and Hospital, National Clinical Research Center for Cancer, Tianjin, China.; 5 The Second Surgical Department of Breast Cancer Oncology, Tianjin Medical University Cancer Institute and Hospital, Key Laboratory of Breast Cancer Prevention and Therapy, National Clinical Research Center for Cancer, Tianjin, China.

**Keywords:** Triple-negative breast cancer, SENP-1, deSUMOylation, GATA-1, CSN5, ZEB1

## Abstract

TNBC is characterized by high incidence of visceral metastasis and lacks effective clinical targets. This study aims to delineate the molecular mechanisms of SENP1 in TNBC invasion and metastasis. By using IHC to test the SENP1 expression in TNBC tissues, we analyzed the relationship between SENP1 expression and TNBC prognosis. We showed that SENP1 expression was higher in TNBC tumor tissues and related to TNBC prognosis, supporting SENP1 as an independent risk factor. High expression of SENP1 was significantly associated with histologic grade and tumor lymph node invasion. Intriguingly, the expression levels of SENP1 in TNBC tumors were significantly correlated with that of CSN5, GATA1 and ZEB1. Importantly, SENP1 promoted TNBC cell migration and invasion by regulating ZEB1 deubiquitination and expression through CSN5. Further studies showed that deSUMOylation at lysine residue K137 of GATA1 enhanced the binding of GATA1 to the CSN5 promoter and transactivated CSN5 expression. In addition, we showed that ZEB1 is deubiquitinated at lysine residue K1108. Our *in vivo* studies also indicated that reduction in SENP1 expression upregulated GATA1 SUMOylation, and thus resulted in decreased expression of CSN5 and ZEB1 in the tumor microenvironment, which decelerated TNBC progression and metastasis. SENP1 promoted CSN5-mediated ZEB1 protein degradation via deSUMOylation of GATA1, and thus influenced TNBC progression. These findings suggest that SENP1 could be utilized as a potential target for blockade of TNBC development and thus provide a totally new approach for TNBC treatment.

## Introduction

Breast cancer is the most common malignancy and a leading cause for death in women 1,2. Based on the expression levels of estrogen receptor (ER), progesterone receptor (PR) and human epidermal growth factor receptor 2 (HER2), breast cancer patients could be classified into 4 subtypes: Luminal A, Luminal B, HER2 enriched and triple negative (TN). TNBC is a special type compared with the others, characterized by high degree of invasion, high occurrence rate in early stage, high visceral metastasis and poor prognosis. Moreover, few targets have been identified for TNBC and thus TNBC patients could not benefit from endocrine or targeted therapies 3-7. Once diagnosed, less than 30% of TNBC patients could survive longer than 5 years 8. Thus, it is important to develop more specific and effective treatment solutions for different breast cancer patients based on their gene expression signatures 9,10.

SUMOylation (small ubiquitin-like modifier) is one type of post-translational modifications that could regulate protein activity and function 11,12. Its reverse process is called deSUMOylation, which is catalyzed by a set of SUMO-specific proteases (SENPs) belonging to the C48 cysteine protease family and possessing highly conservative C-terminal catalyzing domain. These enzymes participate through hydrolase activity in the reaction of SUMO protein maturation, which involves the removal of a short fragment on the C-terminus of SUMO inactive form and exposure of two glycine residues. Based on amino acid sequence homology, intracellular location, and substrate specificity, SENPs are classified into 3 families: Ulp/SENP, Desi, and USPL1, which could be further categorized into 6 subtypes 13-16. These specific cysteine proteases have important functions in SUMO processing and maturation, and in deSUMOylation modification. Previous studies demonstrated that SENPs expression are deregulated in many types of cancer and suggested that this change might relate to tumorigenesis via regulation of SUMOylation 17. Among SENPs, SENP1 (sentrin-specific protease 1) is the most common SUMO-specific peptidase that catalyzes substrate deSUMOylation 18-20. SENP1 is highly expressed in malignant tissues such as in prostate and breast tumors 21. Compared with Luminal A/B and HER-2 enriched subtypes, SENP1 is highly expressed and significantly related to tumor proliferation and invasion in TNBC 22. However, the underlying mechanisms for SENP1 in this process are not clear.

GATA1 (GATA binding protein 1) is a member of the GATA family of transcription factors, and contains 2 zinc finger structure Cys-X2-Cys, a C-terminal zinc finger that could bind to GATA sequence, and an N-terminal zinc finger that could bind to GATC sequence. C-terminal zinc finger could bind to DNA while N-terminal zinc finger could enhance this binding, where they function together to regulate transcription 23. Previous studies suggested that GATA1 is a transcription factor specific for the hematopoietic system. However, recent studies indicated that GATA1 also has an important role in solid tumors. For example, GATA1 is highly expressed in breast cancer 24 and involved in breast cancer epithelial-mesenchymal transition (EMT) process, promoting its invasion and metastasis 25-27. It was reported that SENP1 could enhance downstream gene transcriptional regulation via mediating GATA1 deSUMOylaiton 28. However, the underlying mechanisms for GATA1 deSUMOylation in breast cancer invasion and metastasis are unknown.

According to relevant literatures, we found that GATA1 could regulate transcription of JAB1/CSN5 (COPS5) 29. Coincidentally, COP9 signalosome complex subunit 5 (CNS5) is a deubiquitinating enzyme that could mediate deubiquitination in certain proteins. There are also studies showing that silencing of CSN5 could inhibit the propagation, invasion, and metastasis capacity of TNBC 30, suggesting that CSN5 might have a critical role in TNBC development. It is well known that EMT could promote tumor invasion and metastasis, and ZEB1 (Zinc finger E-box-binding homeobox) is a significant marker for the EMT process. Previous studies displayed that CSN5 could affect ZEB1 protein stability and the EMT process during tumor development via regulation of ZEB1 deubiquitination 31. In breast cancer, ZEB1 expression also correlates with EMT 32,33.

In this study, we explored the relationship between SENP1 expression and TNBC metastasis, using TNBC tumor tissues as well as cell lines. Our study indicated that SENP1 could regulate GATA1 deSUOMOylation and reduce GATA1 SUMOylation, and thus downregulate GATA1 transcriptional activity, which further reduces CSN5 transcription and expression. Reduced expression of CSN5 could lead to deubiquitination of a key EMT regulator, ZEB1, which further reduces EMT and thus inhibits the invasion and metastasis abilities in TNBC patients.

## Materials and methods

### Chemicals and antibodies

Fetal bovine serum (FBS) (Hyclone, Logan, UT, USA); Leibovitz's L-15 culturing medium (GIBCO, Invitrogen Corporation, NY, USA); RPMI 1640 culturing medium (GIBCO, Invitrogen Corporation, NY, USA); Trypsin-EDTA solution (Gibco, Grand Island, NY, USA); G418 (Sigma, St. Louis, MO, USA); Crystal violet staining solution (KeyGEN Biotech, NanJing, China); Ready-to-use immunohistochemistry kit (Maxim, Fuzhou, China); HE staining kit (KeyGEN Biotech, NanJing, China); QuickMutationPlus Site-Directed Mutagenesis Kit (Beyotime, ShangHai, China); pGL3 Luciferase Reporter Vector (Promega Corporation, London, UK); Luciferase Assay Reagent (Promega, Madison, USA); Matrigel Matrix (BD Bioscience, USA); Imprint Chromatin Immunoprecipitation Assay kit (Millipore, Burlington, Massachusetts, USA); RT-PCR kit (Takara, Japan); Trizol (Takara, Japan); iScript cDNA Synthesis Kit (Bio-Rad, Hercules, CA, USA); SYBR Green Premix Ex TaqTM (Takara, Japan); Diethyl pyrocarbonate (DEPC) (Amresco, Ohio, USA); PCR primers were synthesized by Sangon Biotech (Sangon, ShangHai, China); PCR purification kit (Takara, Japan); T4 DNA ligase and restriction enzymes (NotI, XbaI, AgeI, EcoRI) (Takara, Japan); Plasmid Purification Kit (Takara, Japan).

Anti-SENP1 antibody (Abcam, ab108981); Anti-E-Cadherin antibody (Abcam, ab40772); Anti-Keratin12/K12 antibody (Abcam, ab185627); Anti-Vimentin antibody (Abcam, ab92547); Anti-Survivin antibody (Abcam, ab76424); Anti-SNAIL antibody (Abcam, ab216347); Anti-SLUG antibody (Abcam, ab51772); Anti-Twist antibody (Abcam, ab175430); Anti-ZEB1 antibody (Abcam, ab155249); Anti-ZEB2 antibody (Abcam, ab138222); Anti-GATA1 antibody (Abcam, ab181544); Anti-SMURF1 antibody (Abcam, ab94480); Anti-SYVN1/HRD1 antibody (Abcam, ab225891); Anti-Sumo 1 antibody (Abcam, ab133352); Goat Anti-Rabbit IgG H&L(HRP) (Abcam, ab6721); Rabbit Anti-Mouse IgG H&L(HRP) (Abcam, ab6728); Anti-MDM2 (D1V2Z) antibody (CST,#86934); Anti-C/EBPbeta antibody (CST, #3087); Anti-GAPDH antibody (CST, #5174); Anti-Flag antibody (CST, #2908); Anti-USP51 (CENTER) antibody (Millipore, SAB1305451).

### Cell lines and cell culturing

BT549, HCC1143, HCC1937 and MDA-MB-231 cell lines were purchased from the American Tissue Culture Collection (ATCC, Rockville, MD, USA). All the cell lines were cultured in RPMI 1640 culturing medium supplemented with 10% FBS, in 37 ℃ incubator with 95% humidity and 5% CO_2._

### Real-time quantitative reverse transcription (qRT) PCR

Total RNA was extracted using TRIzol according to the instruction (Invitrogen), and was reverse transcribed using a reverse transcription kit from Takara. 1 μg cDNA was mixed with the primers and SYBR Green PCR Master Mix (TaKaRa) for real-time qRT-PCR reaction. 3 technical repeats were done for each sample. PCR primers used were listed in supplementary table S1.

### Western blotting analysis

Tissues or cells were lysed by RIPA buffer supplemented with PFMS and protease inhibitor mixture (Invitorgen). The lysed samples (20 μg) were applied to SDS-PAGE for protein separation, followed by Western blotting to detect target proteins. Antibodies used were listed in Supplementary table S2.

### Wound-healing and Transwell invasion assays

Cells were cultured in 6-well plates in 37 ℃ incubator and PBS supplemented with mitomycin was added into each well. A 10-μL tip was used to vertically scratch a line to evaluate wound closure. After scratching, PBS solution was removed, and Leibovitz's L-15 medium was added for further culturing in 37 ℃ incubator. The line positions were recorded under microscopy at 0 and 72 h respectively after scratching. Image J software was applied to measure the wound areas, and recovery rate of the areas was calculated at 72 h. The experiment was repeated for 3 times.

After addition of 40 μL Matrigel to the 24-well Transwell, and solidification of the gel in a 37 ℃ incubator for 15-30 minutes, 200 μL suspended cell solution (2.5×10^5^ cell/ml) was added on the gel carefully. 600 μL Leibovitz's L-15 medium supplemented with 10% FBS and specific chemo-attractants was added to the bottom of the lower chamber of the 24-well plate. The cells were cultured in a 37 ℃ incubator for 24 hours. After that, 700 μL of 1% crystal violet solution was added into the bottom wells. Inverted microscopy was then used to count cells that passed the gel in the Transwell. The counting areas were randomly chosen, and the experiment was repeated for 3 times.

### Plasmid constructs, gene expression alteration and determination

Targets were chosen for the aimed sequence of SENP1 according to RNAi designing principle, and primers containing the target sequence as well as desired enzyme cutting sites were synthesized. The amplified sequences were cloned into the shRNA lentiviral vectors. 0.5×10^5^ MDA-MB-231 cells were seeded into 24-well plates and cultured in 37 ℃ incubator with 5% CO2. After the cells reached 90% confluency, culturing medium was replaced with 0.5 ml Polybrene/medium mixture and 20μL lentivirus expression vectors were added to each well under specific MOI values. After culturing for another 48 hours, medium was replaced with new medium containing 200 μg/mL Puromycin for single clone selection.

According to the sequences of CSN5, GATA1 and ZEB1 and multiple cloning sites of the CMV-C-Flag vectors, specific primers containing upstream and downstream restriction sites (NotI: GCGGCCGC; XbaI: TCTAGA, respectively) were designed and synthesized. Coding sequences of CSN5, GATA1 and ZEB1 genes were amplified and purified. Lentiviral expression vectors were constructed for wild-type GATA1, ZEB1 and CSN5. The constructed vectors were validated using PCR. Same transfection methods were used as above where however medium containing 200 μg/ml G418 were used for single clone selection.

Lentiviral vectors expressing point-mutated proteins of GATA1 (K137R) and ZEB1 (K1108A, K186A, K439A) were constructed using respective primers. Same methods were used for cell transfection and selection as above (G418). Cells were collected at indicated time points for further experiments. Primers used were listed in supplementary Table S3.

### Patient tissues and Immunohistochemistry

All aspects of the study were approved by the Ethic Committee of the Tianjin Medical University General Hospital. Surgical dissected tumor samples and adjacent normal tissues as well as the pathological reports were received from the Hospital. Patients' pathological and clinical characteristics were listed in Table 1. SENP1 and CSN5 expression levels in TNBC samples were detected using IHC with a DAB kit. Semi-quantitative immunoreactive score (IRS) was used to estimate the ratio of SENP1 positive tumor cells. 5 random areas were chosen under the light microscopy, where staining intensity was scored 0 (negative), 1 (low expression), 2 (medium expression) or 3 (high expression), and the positive cell proportion was scored 0 (0%), 1 (1-25%), 2 (26-50%) or 3 (51-100%). The IRS score was determined by the multiplication between the positive cell proportion score and the staining intensity score, which ranges from 0-9. A score <2 was marked as negative (-), a score between 2 to 3 marked as low expression (+), a score between 4 to 6 marked as medium expression (++), and a score higher than 6 marked as high expression (+++). Another cohort of 1099 breast cancer patients with mRNA expression profiling were included from The Cancer Genome Atlas (TCGA) database.

### Chromatin immunoprecipitation assay (ChIP)

ChIP was carried out using a commercial kit (Millipore, #CHP1-24RXN, Burlington, Massachusetts, USA) according to the manual. The results were further validated and analyzed by PCR.

### Co-immunoprecipitation (co-IP)

Cells were lysed in RIPA buffer (Thermo Scientific, Pittsburgh, PA, USA) for 30 minutes at 4 °C, followed by centrifugation at 13,000 g. The supernatant was transferred to a new tube and put for another 30 minutes at 4 °C. 40 μl protein A/G agarose beads (Calbiochem, #IP05) was added to the tube for pre-incubation for 30 minutes at 4 °C. The tubes were centrifuged, and supernatant was collected. Desired antibodies were added into the supernatant and the tubes were rotated and incubated for overnight at 4 °C. The next day protein A/G agarose beads were added to the incubated solution and tubes were rotated and incubated for another 4 hours at 4°C. After washing, beads were collected by centrifugation and mixed with loading buffer, followed by SDS-PAGE protein separation and immunoblotting.

### Luciferase reporter assay

Cells were transfected with respective plasmids in 96-well plates. After 48 hours, 100 μl lysis buffer was added into each well for adequate lysis. After centrifugation, supernatant was taken for following measurement. Firefly luciferase and related reagents were dissolved and the time interval for the automatic ELIZA analyzer was set to 2 s, and measure time was set to 10 s. Reagents were added into each sample tube and the relative light units (RLU) was measured after mixture.

### Tumour xenograft study *in vivo*

For Breast tumor lung metastasis model, 0.2 ml cell suspensions (1×10^6^/mL) were intravenously injected into nude mouse tails. 6 mice were used for each group. 30 days after the injection, the lungs were collected, and their weight was measured. The lung tissues were embedded in paraffin and sectioned. The paraffin sections were deparaffinized in xylene, rehydrated with gradient ethanol, stained by hematoxylin and eosin, dehydrated in gradient ethanol, cleared by xylene and sealed in neutral balsam. Random areas were chosen under phase contrast microscopy at 400 x magnification and metastasized tumors were counted in each sample.

For Subcutaneous tumor model, 0.2 ml cell suspensions (5×10^7^/mL) were subcutaneously injected into the left flank of nude mice. 8 mice were used for each group. Mouse body weight and tumor size were measured every 3 days. Tumor size was calculated using the formula: tumor volume (TV) = 1/2×a×b^2^, where a and b represent the length and width of the tumors respectively measured by caliper. 30 days after the injection, tumors were dissected out and embedded in paraffin for further experiments.

### Statistics

All data are presented as means ± SD. All experiments were repeated 3 times independently. Statistical analysis was performed with paired 2-tailed Student's *t* test. ANOVA was used for continuous variables of two groups. Categorical variables were analyzed by Fisher or Chi-square tests. Survival curve is analyzed by Kaplan-Meier method and compared statistically using the log-rank test in SPSS (IBM, 22.0 version). *P* < 0.05 is regarded as statistically significant.

## Results

### SENP1 is highly expressed in majority of TNBCs and a poor prognosis is related to high SENP1 expression

**Figure 1:**
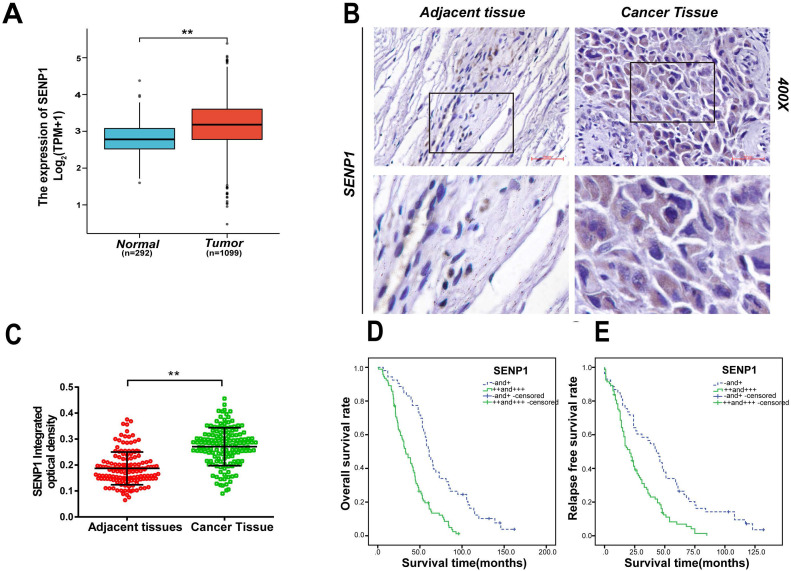
**The expression of SENP1 in TNBC and adjacent normal tissues. A** TCGA show the expression of SENP1 in breast cancer tissue(n=1099) and adjacent normal tissues(n=292) (*P*< 0.001). **B, C** The expression of SENP1 were in TNBC tissue (n=146) and adjacent tissues (n=146) detected by IHC. Results were shown as mean ± SD. **D, E** Overall survival (OS) and relapse-free survival (RFS) are significantly different according to the expression level of SENP1 in TNBCs. The result was analyzed with t test and log-rank test. *P < 0.05, **P < 0.01.

From the Cancer Genome Atlas (TCGA) datasets, we found the expression for SENP1 is significantly increased in breast tumors compared with adjacent normal tissues (BC 1099 cases vs adjacent normal 292 cases, *P* < 0.001) (Fig.1A) 34. To further understand the role of SENP1 in TNBC development, we used IHC to detect expression of SENP1 in these patients. In the 146 TNBC cases, no patients received neoadjuvant chemotherapy. SENP1 signal was detected in majority (87%) of the TNBC samples (Table 1). According to IHC staining in 146 TNBCs, SENP1 is significantly higher expressed in tumor tissues compared with adjacent normal tissues (*P* < 0.001) (Figs. 1B,C). In addition, SENP1 expression was positively correlated with histologic grade (*P* = 0.045, χ2 = 6.918, r = 0.202) and lymph node metastasis (*P* = 0.023, χ2 = 9.564, r = 0.256) in TNBC specimens (Table 1). To further explore the role of SENP1 in TNBC development, we also did univariate and multivariate analyses of clinicopathologic factors for the follow-up data in the TNBC cohort (Table 2). Moreover, overall survival (OS) and relapse-free survival (RFS) were significantly lower in TNBC SENP1 high (+++) or medium (++) expression groups, compared with negative (-) or low expression (+) groups (*P* < 0.001; OS: 33 and 61 months, respectively; RFS: 21 and 43 months, respectively) (Figs. 1D,E). SENP1 expression is an independent risk factor for TNBC development.

### Reduced SENP1 inhibits TNBC cancer cell invasion and metastasis via downregulation of ZEB1 expression shown by *in vitro*

**Figure 2:**
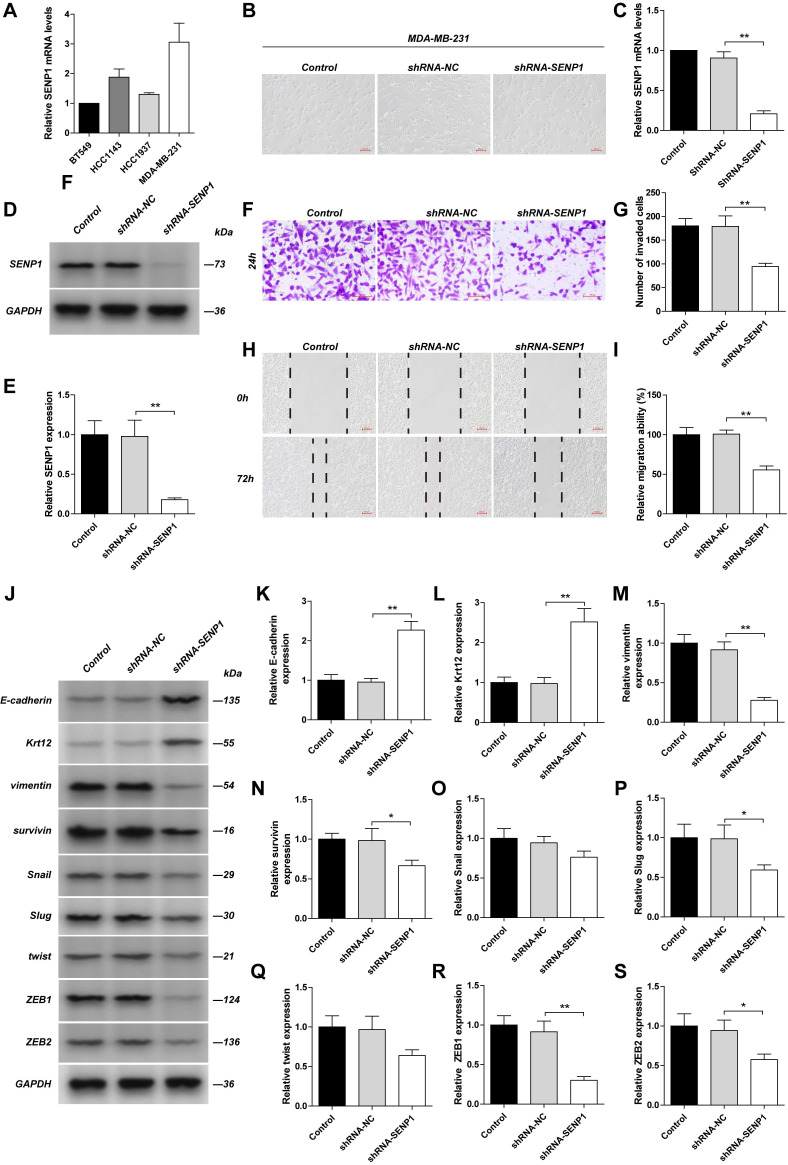
**The effect of SENP1 on EMT pathway in TNBC cells**. The MDA-MB-231 cells was transfected with shRNA-NC or shRNA-SENP1 by lentiviral vector. SENP1 in MDA-MB-231 cells were stably knockdown by lentiviral vector. **A** The mRNA levels of SENP1 in BT549 cells, HCC1143 cells, HCC1937 cells, MDA-MB-231 cells was detected by qPCR. **B** The morphology of the MDA-MB-231cells was observed with a light microscope.** C, D, E** The efficiency of SENP1 knockdown were measured by WB and qPCR. **F, G** MDA-MB-231 cells were seeded on chambers for 24 h. Cells that migrated through the matrigel-coated chambers were stained with crystal violet. Representative images were captured, and the cells were counted from three independent experiments. **H, I** The lateral migration ability was measured by wound healing assays. Representative images of wound were captured at 0 h and 72 h, the healed rate is presented. **J** Western blot analysis of EMT pathway-related proteins as E-cadherin (**K**), Krt12 (**L**), Vimentin (**M**), Survivin (**N**), Snail (**O**), Slug (**P**), Twist (**Q**), ZEB1 (**R**), ZEB2 (**S**) in different group. The Results were shown as mean ± SD.*P < 0.05, **P < 0.01.

By detecting mRNA levels in several TNBC cancer cells (BT549, HCC143, HCC1937, MDA-MB-231), we found that SENP1 mRNA level is highest in MDA-MB-231cells (Fig. 2A). Downregulation of SENP1 in MDA-MB-231 cells reduced their invasion and metastasis abilities, and resulted in a round cellular shape (Fig. 2B-I). Expression of epithelial cell associated proteins, such as E-Cadherin and Krt12, were significantly increased, while expression of mesenchymal cell associated proteins, including Vimentin, Survivin, Slug, ZEB1 and ZEB2, were significantly down-regulated, in these cells. Expression of Snail and Twist decreased as well but is not statically significant. ZEB1 showed the most significant change (Fig. 2J-S). The results indicate that SENP1 downregulation blocks epithelial-mesenchymal transition (EMT) process and thus inhibits breast cancer cell invasion and metastasis, which is mediated via ZEB1 expression.

### SENP1 regulates ZEB1 expression via regulation of the ubiquitinating enzyme CSN5

**Figure 3:**
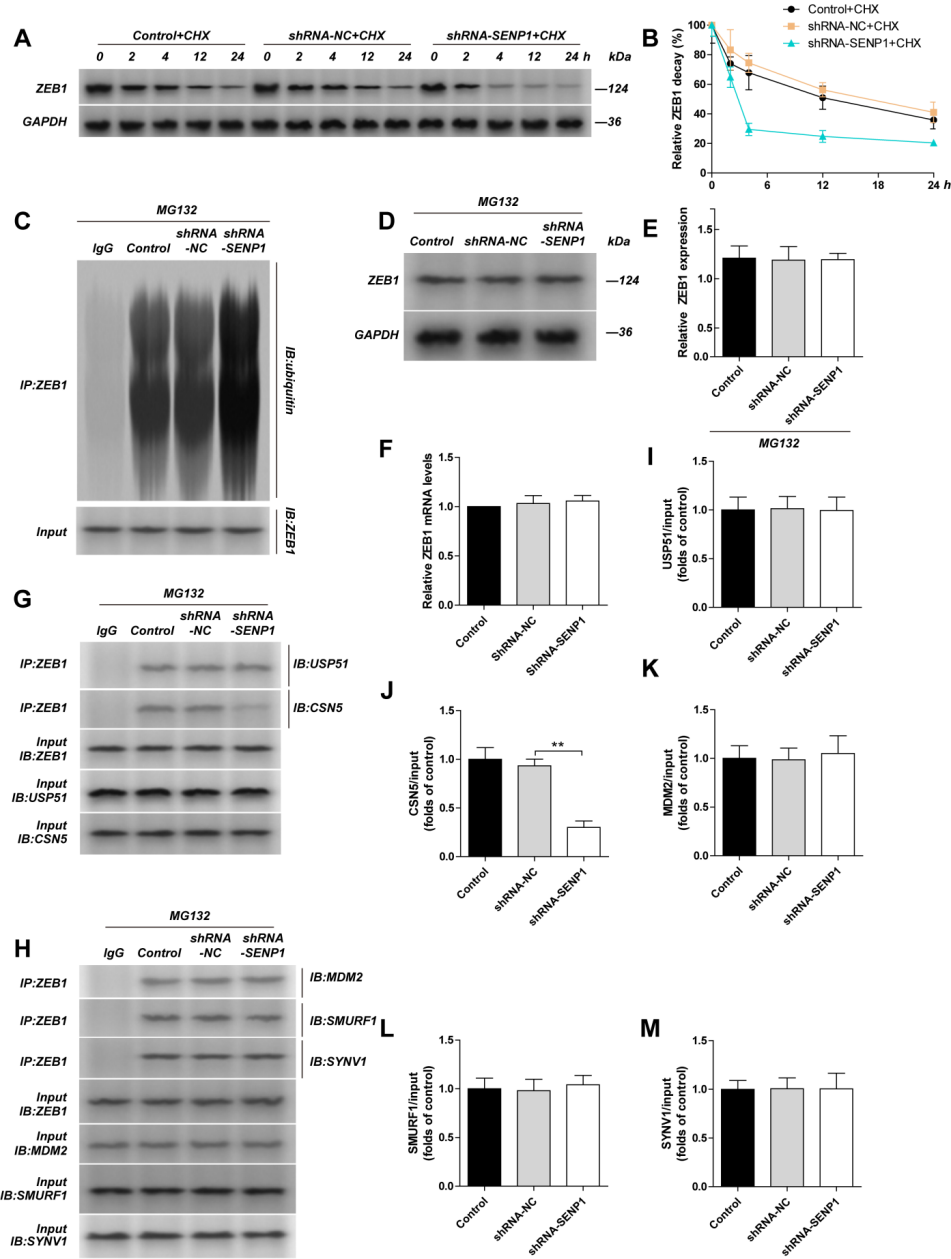
**Knockdown SENP1 inhibit ZEB1 ubiquitylation**. The MDA-MB-231 cells was transfected with shRNA-NC or shRNA-SENP1 by lentiviral vector. A, B MDA-MB-231 cells, shRNA-NC-MDA-MB-231 cells, shRNA-SENP1-MDA-MB-231 cells were treated with CHX (10μg/ml) for 0, 2, 4, 12, 24 h. The half-life of ZEB1 was detected by western blot assay. **C, D, E** MDA-MB-231 cells, shRNA-NC-MDA-MB-231 cells, shRNA-SENP1-MDA-MB-231 cells were treated with MG132 (10 μM) for 24 h, and then the ZEB1 levels were detected by western blot assay. The cell extracts from the indicated groups were subjected to immunoprecipitation with anti-ZEB1 antibody or anti-IgG antibody, followed by immunoblot with anti-ubiquitin antibody. **F** ZEB1 mRNA levels were detected by qPCR in different groups transfected with shRNA-NC or shRNA-SENP1 by lentiviral vector. **G, H** The cell extracts from the indicated groups were subjected to immunoprecipitation with anti-ZEB1 antibody or anti-IgG antibody, followed by immunoblot with anti-USP51 antibody (**I**), anti-CSN5 antibody (**J**), anti-MDM2 antibody (**K**), anti-SMURF1 antibody (**L**) or SYNV1 antibody (**M**). Results were mean ± SD for three individual experiments which, for each condition, were performed in triplicate. *P<0.05, **P<0.01.

When we used cycloheximide (CHX) to inhibit protein synthesis, by detecting ZEB1 protein level at indicated time points (Fig. 3A, B), we found that the half-life of ZEB1 reduced, and its degradation was enhanced. To study whether the change in ZEB1 was related to proteasomes, we used MG132 to treat the cells and measured ZEB1 protein level again, and found that the difference in ZEB1 protein levels between shRNA-NC and shRNA-SENP1 groups were gone. We further detected the ubiquitination in ZEB1 using Co-IP and found that ZEB1 ubiquitination was significantly increased in the shRNA-SENP1 group (Fig. 3C, D, E). However, mRNA level of ZEB1 was not changed after the downregulation of SENP1 (Fig. 3F). In addition, we also checked the binding affinity of ZEB1 with ubiquitinating enzymes, including USP51 and CSN5, and E3 ligases, including MDM2, SMURF1 and SYVN1, after downregulation of SENP1 (Fig. 3G-H). The results indicated that ZEB1 binding with USP51, CSN5, MDM2, SMURF1 and SYVN1 was not affected, however, the binding affinity between ZEB1 and CSN5 was reduced (Fig. 3I-M). After knocking down SENP1 in MDA-MB-231 cells, the invasion capacity of these cells was significantly reduced. However, when SENP1 was knocked down, invasion capacity of these cells was significantly upregulated. Further, overexpression of CSN5 reversed the effect of SENP1 in MDA-MB-231 cells (Supplementary Fig. 1).

### SENP1 regulates CSN5 transcription through deSUMOylation of GATA1

**Figure 4:**
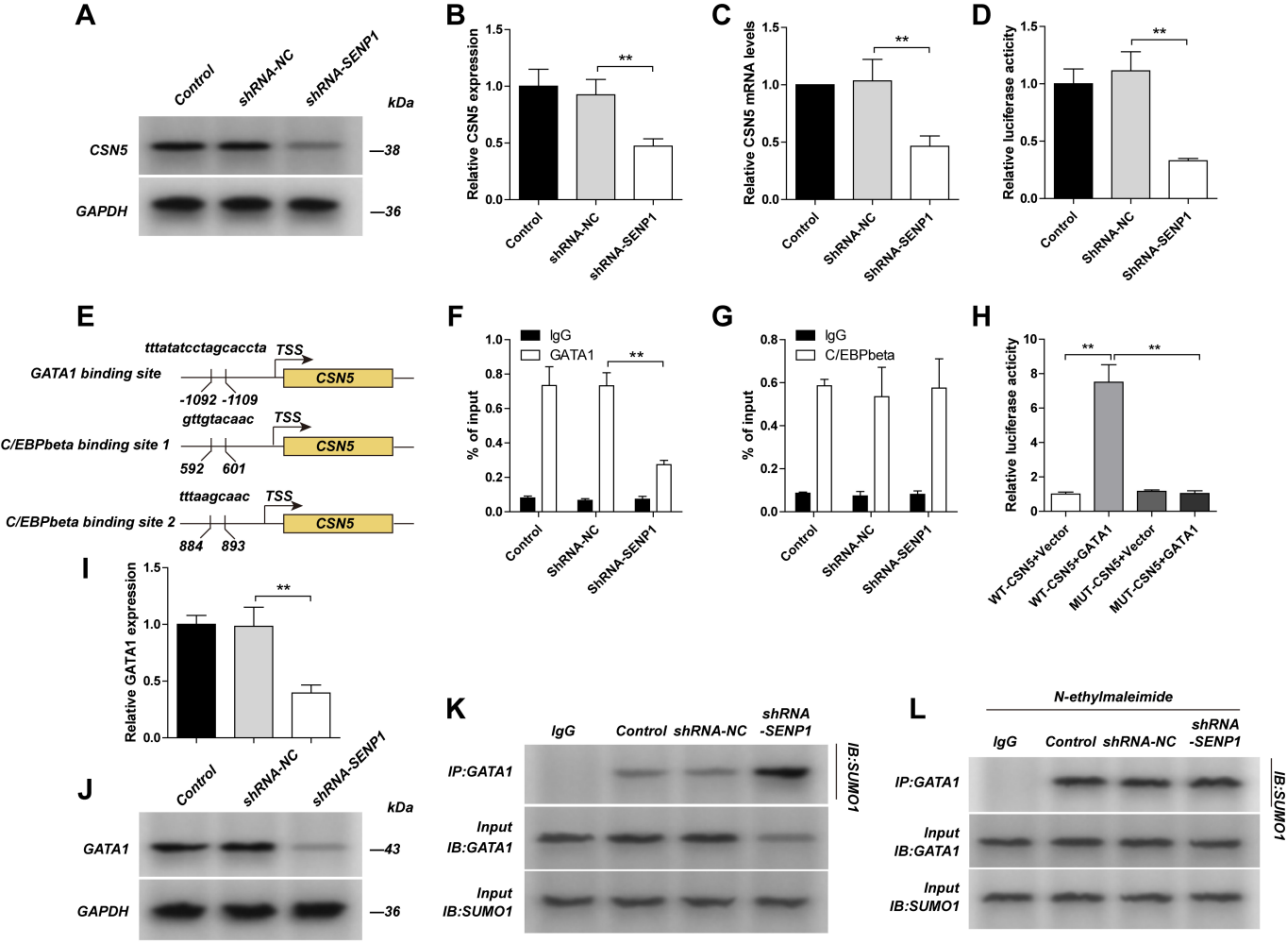
** Knockdown SENP1 inhibited CSN5 expression**. **A,B,C** The MDA-MB-231 cells was transfected with shRNA-NC or shRNA-SENP1 by lentiviral vector. The expression of CSN5 and The mRNA levels of CSN5 was measured by qPCR. **D** The promotor activity of CSN5 was measured by luciferase reporter assay. **E** Binding site of GATA1 and C/EBPbeta with the promoter of CSN5. **F, G** GATA1 or C/EBPbeta interacting with the promoter region of CSN5 was determined by Chip assay. **H** GATA1 directly regulates CSN5 in The HEK293 cells which transfected withWT-CSN5+Vector, WT-CSN5+GATA1, MUT-CSN5+Vector or MUT-CSN5+GATA1 by Dual Luciferase Reporter Assay. **I, J** GATA1 were detected by western blot assay. **K** The cell extracts from the indicated groups were subjected to immunoprecipitation with anti-GATA1 antibody or anti-IgG antibody, followed by immunoblot with anti-SUMO1 antibody. **L** MDA-MB-231 cells, shRNA-NC-MDA-MB-231 cells, shRNA-SENP1-MDA-MB-231 cells were treated with N-ethylmaleimide (1 mM) for 24 h. The cell extracts from the indicated groups were subjected to immunoprecipitation with anti-GATA1 antibody or anti-IgG antibody, followed by immunoblot with anti-SUMO1 antibody. Results were mean ± SD for three individual experiments which, for each condition, were performed in triplicate. * P <0.05, ** P <0.01.

We detected CSN5 protein level, mRNA level, its promotor activity and promotor binding affinity with transcription factors (GATA1 and C/EBPbeta) in SENP1 downregulated cells, and found that the protein level, mRNA level and promotor activity of CSN5 were significantly decreased; binding affinity for CSN5 promotor with GATA1 was decreased, while for C/EBPbeta was unchanged, indicating SENP1 regulates CSN5 expression via transcriptional regulation of CSN5 (Fig. 4A-G). As was showed one GATA1 binding site and two C/EBPbeta binding sites in the CSN5 promoter, corresponding to the transcription start site (TSS). In HEK293 cells, compared with the WT-CSN5+ Vector group, fluorescence intensity of the WT-CSN5+ GATA1 group was significantly increased, while the MUT-CSN5+ Vector group had no significant change. Compared with the WT-CSN5+Vector group, fluorescence intensity of the MUT-CSN5+ GATA1 group did not change significantly. These results indicated that GATA1 can directly up-regulate the transcriptional activity of CSN5 in dual luciferase reporter assay (Fig. 4 H). We detected GATA1 protein level and its SUMOylation in SENP1 downregulated cells, and found that GATA1 protein level was decreased while SUMOylation was enhanced (Fig. 4I-K). To further study how SENP1 affects GATA1 SUMOylation, we used N-ethylmaleimide (NEM), a SUMO protease inhibitor, to treat the cells in different groups and found that there was no difference for GATA1 SUMOylation in these groups (Fig. 4L). All these results indicate that SENP1 regulates GATA1 expression by affecting its SUMOylation status.

### SENP1 regulates GATA1 expression via SUMOylation at lysine residue K137 of GATA1

**Figure 5:**
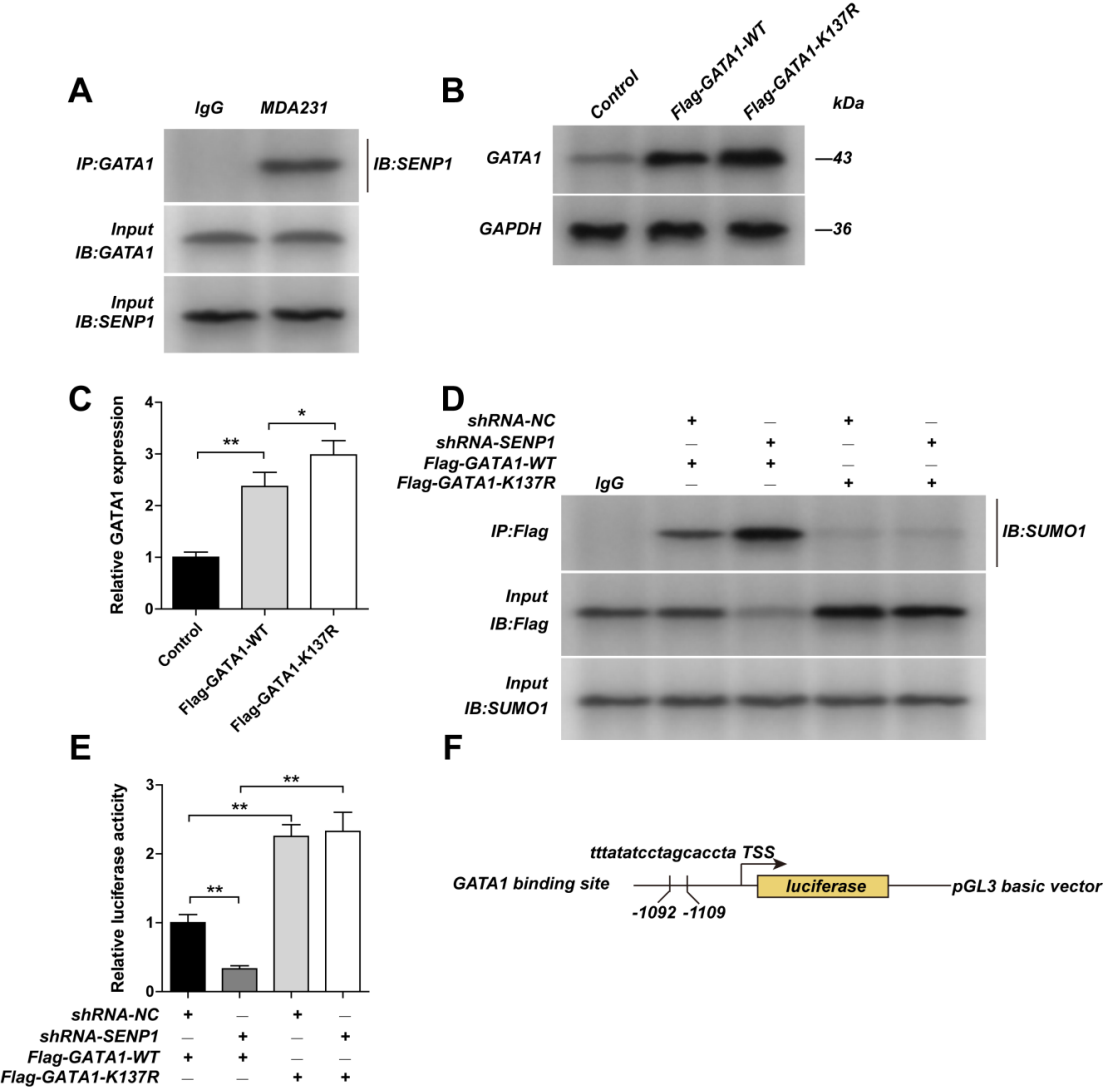
**GATA1 K137R mutants impaired sumoylation of GATA1. A** The MDA-MB-231 cell extracts from the were subjected to immunoprecipitation with anti-GATA1 antibody or anti-IgG antibody, followed by immunoblot with anti-SENP1 antibody. The MDA-MB-231 cells were transfected with Flag-GATA1-WT plasmid or Flag-GATA1- K137R plasmid by lentiviral vector. **B, C** The expression of GATA1 were detected by western blot assay. ShRNA-NC-MDA-MB-231 cells, shRNA-SENP1-MDA-MB-231 cells were transfected with Flag-GATA1-WT plasmid or Flag-GATA1- K137R plasmid by lentiviral vector. **D** And the cell extracts from the indicated groups were subjected to immunoprecipitation with anti-GATA1 antibody or anti-IgG antibody, followed by immunoblot with anti-SUMO1 antibody. **E** The promotor activity of CSN5 was measured by luciferase reporter assay. **F** Binding site of GATA1 with the promoter of CSN5. Results were mean ± SD for three individual experiments which, for each condition, were performed in triplicate. * P <0.05, ** P <0.01.

By using Co-IP, we confirmed that in MDA-MB-231 cells GATA1 could bind to SENP1 (Fig. 5A). We mutated the SUMOylation site of GATA1 by constructing GATA1-K137R expression vectors. We found that expression of GATA1 is higher in GATA1-K137R transfected cells, compared with GATA1-WT transfected cells (Figs. 5B,C). Moreover, we found that in SENP1 downregulated cells, after transfection of GATA1-WT or GATA1-K137R expression vectors, the effects of SENP1 on GATA1 SUMOylation in GATA1-K137R group were totally reversed, showing almost no GATA1 SUMOylation (Fig. 5D). By using dual-luciferase reporter assay (Figs. 5E,F), the inhibition of SENP1 on CSN5 promotor region was released after transfection of GATA1-K137R. These results further confirmed that SENP1 regulates GATA1 expression by affecting its SUMOylation status, which further regulates the expression of CSN5. Overexpressing WT-GATA1 or GATA1-K137R, or knocking-down CSN5 in MDAMB-231 cells significantly reduced their invasion ability, indicating that GATA1 affects the invasion ability of MDA-MB-231 cells by regulating CSN5 expression levels (Supplementary Fig. 2).

### CSN5 regulates ZEB1 expression largely through mediating its deubiquitination of lysine residue K1108

**Figure 6:**
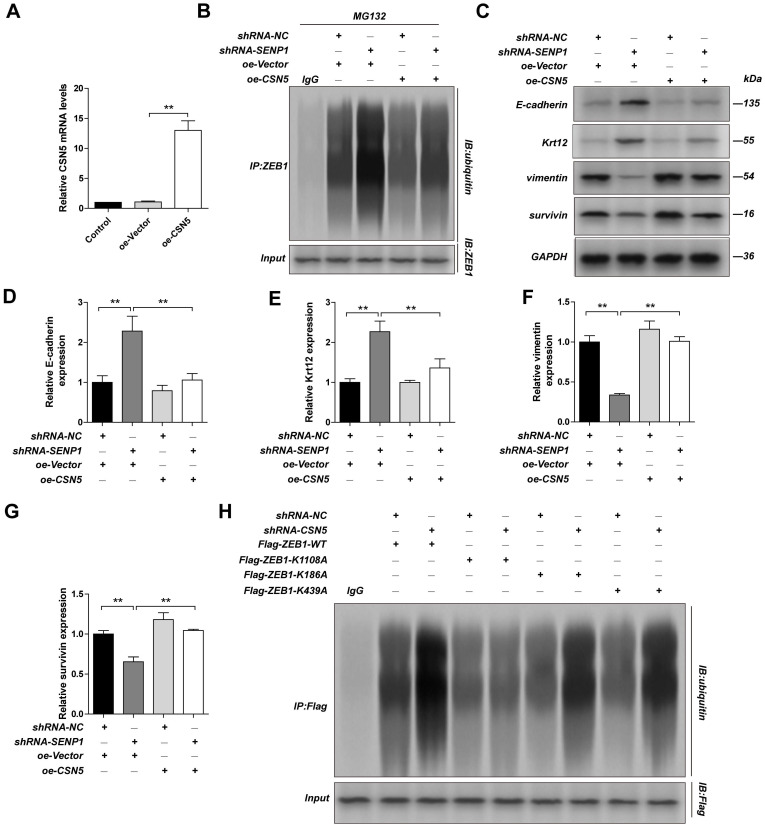
** CSN5 implicate ZEB1 ubiquitination.** The MDA-MB-231 cells were transfected with oe-Vector plasmid or oe-CSN5 plasmid by lentiviral vector. **A** The efficiency of CSN5 overexpression were measured by qPCR. ShRNA-NC-MDA-MB-231 cells, shRNA-SENP1-MDA-MB-231 cells were transfected with oe-Vector plasmid or oe-CSN5 plasmid by lentiviral vector. **B** And the cell extracts from the indicated groups were subjected to immunoprecipitation with anti-ZEB1 antibody or anti-IgG antibody, followed by immunoblot with anti-ubiquitin antibody. **C, D, E, F, G** The expression of E-cadherin, Krt12, Vimentin, Survivin in different group were detected by western blot assay. MDA-MB-231 cells were transfected with ShRNA-NC+Flag-ZEB1-WT, ShRNA-CSN5+Flag-ZEB1-WT, ShRNA-NC+Flag-ZEB1-K1108A, ShRNA-CSN5+Flag-ZEB1-K1108A, ShRNA-NC+Flag-ZEB1-K186A, ShRNA-CSN5+Flag-ZEB1-K186A, ShRNA-NC+Flag-ZEB1-K439A or ShRNA-CSN5+ Flag-ZEB1- K439A by lentiviral vector. **H** The cell extracts from the indicated groups were subjected to immunoprecipitation with anti-ZEB1 antibody or anti-IgG antibody, followed by immunoblot with anti-ubiquitin antibody. Results were mean ± SD for three individual experiments which, for each condition, were performed in triplicate. * P <0.05, ** P <0.01.

We overexpressed CSN5 in SENP1 downregulated MDA-MB-231 cells and found that CSN5 overexpression could reduce ubiquitination in ZEB1 (Fig. 6A-B) and reverse the effects SENP1 has on the EMT related proteins, including E-Cadherin, Krt12, Vimentin, Survivin (Fig. 6C-G). To explore which deubiquitination site in ZEB1 is regulated by CSN5, we constructed expression vectors for ZEB1-K1108A, ZEB1-K186A and ZEB1-K439A. After transfection of these plasmids in CSN5 downregulated MDA-MB-231 cells, we showed that ZEB1 ubiquitination was significantly lower in MDA-MB-231 cells, compared with other groups, indicating that CSN5 mediates ZEB1 deubiquitination at K1108 (Fig. 6H). These results demonstrated that SENP1 might regulate EMT via regulating GATA1 SUMOylation, which further affects CSN5 expression, ZEB1 ubiquitination and finally affects ZEB1 expression.

### SENP1 regulates GATA1 SUMOylation, CSN5 and ZEB1 expression and tumor development and metastasis shown by *in vivo*

**Figure 7:**
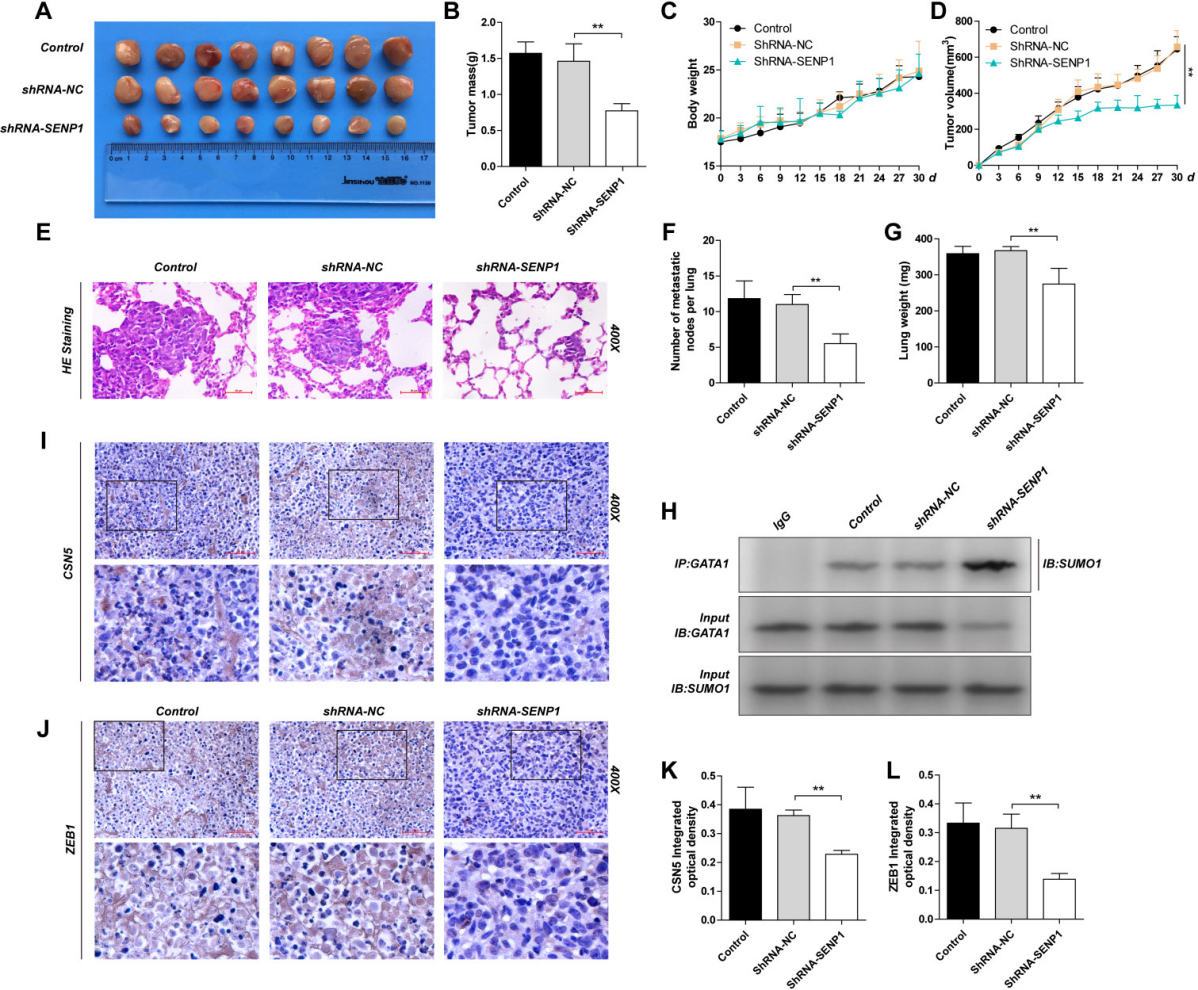
** The effect of SENP1 on the tumorigenicity and metastatic of MDA-MB-231 cells *in vivo*. A** The tumors of mice were excised from the indicated groups after indicated treatment. **B** The tumor masses for two groups of animals were compared, and each histogram represented the mean ± S.D. of 8 mice. **C** The mouse body weight was measured every 3 days. **D** The tumor volumes of the indicated groups were measured and calculated once every 3 days. **E** The lung weight. **F** Representative HE staining of lungs separated from the nude mice which lung metastasis model. and each histogram represented the mean ± S.D. of 6 mice. **G** Number of metastatic nodes per lung was been counted. **H** The tubecomors tissue extracts from the indicated groups were subjected to immunoprecipitation with anti-GATA1 antibody or anti-IgG antibody, followed by immunoblot with anti-SUMO1 antibody. **I, K** IHC staining of tumor tissue from the indicated groups were detected by ZEB1 antibody. **J, L** IHC staining of tumor tissue from the indicated groups were detected by CSN5 antibody. Results were shown as mean ± SD. * *P* <0.05 ** *P* <0.01.

We established subcutaneous mouse models and lung metastasis nude mouse models using the MDA-MB-231, shRNA-NC-MDA-MB-231 and shRNA-SENP1-MDA-MB-231 cell lines, to explore the role of SENP1 in tumor formation and metastasis *in vivo*. Results showed that SENP1 down-regulation led to significantly decrease in ZEB1 and CSN5 expression (Fig. 7I-L), reduced tumor size and light tumor weight (Fig. 7A, D), slower tumor growth (Fig. 7B), less lung metastasized lymph nodes and reduced lung weight (Fig. 7 E-G), while the overall mouse weight within each group was not significantly changed (Fig. 7C). We conducted co-IP using transplanted tumor tissues and found that in the SENP1 down-regulated group, GATA1 binding affinity with SUMO1 was increased and GATA1 SUMOylation was enhanced as well (Fig. 7H).

### Correlation between expressions of SENP1, CSN5, GATA1 and ZEB1 in TNBC tissues

**Figure 8:**
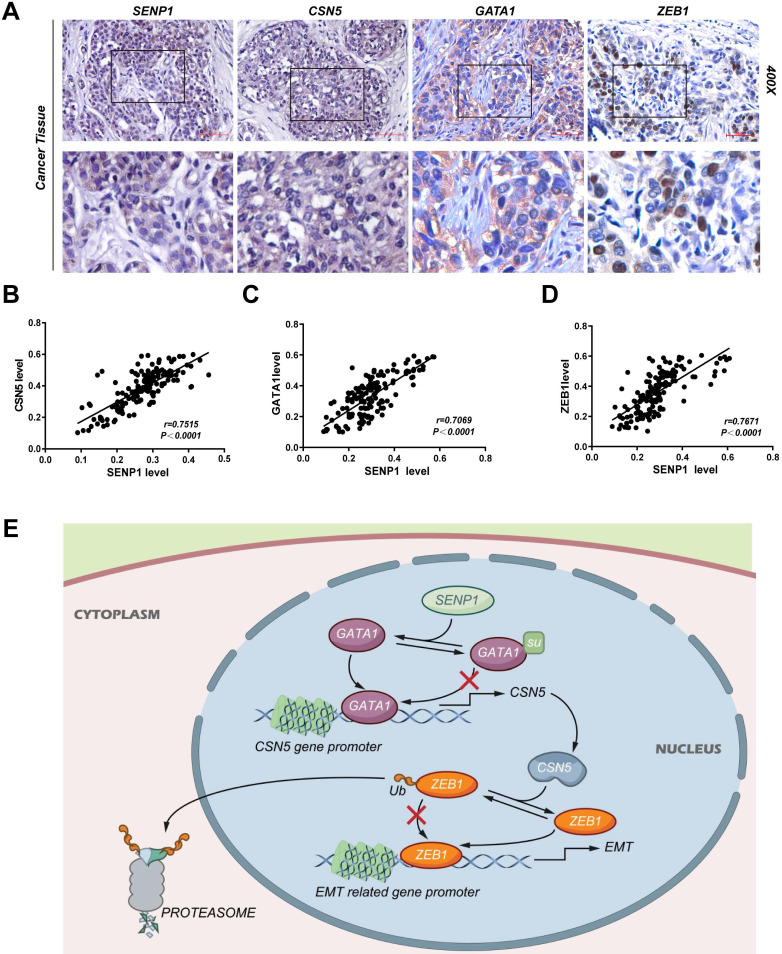
CSN5, GATA1 and ZEB1 is correlated with SENP1 in TNBC tissues. **A** IHC staining of SENP1, CSN5, GATA1 and ZEB1 in TNBC tissue. **B, C, D** The correlation of SENP1 level vs CSN5, GATA1 and ZEB1 level respectively in TNBC(n=146). **E** Graphical abstract.

The expression of SENP1, CSN5, GATA1 and ZEB1 in TNBC tissue were detected by IHC (n = 146) (Fig. 8A). Expression of CSN5 (r = 0.7515, *P* < 0.001), GATA1 (r = 0.7069, *P* < 0.001) and ZEB1 (r = 0.7671, *P* < 0.001) was significantly positive correlated with SENP1 expression (Fig. 8 B-D). SENP1 mediated deSUMOylation of GATA1 and enhanced binding of GATA1 to the CSN5 promoter transactivated CSN5 expression. CSN5, which is a deubiquitin ligase for ZEB1, regulated ZEB1 protein stability and thus TNBC cell invasion and metastasis via EMT (Fig. 8E).

## Discussion

In this study, we investigated the function of SENP1 in TNBC invasion and metastasis. We first noticed that SENP is significantly highly expressed in TNBC tumor tissues compared with adjacent tissues 22. In tumors such as prostate cancer, neuroblastoma (NB), and breast cancer, SENP1 is also related to tumor development and metastasis 21,35,36. It is also reported that SENP1 inhibits tumor development via regulation of myeloid-derived suppressor cell (MDSC), which is important in immune suppression 37. More importantly, in this study we found that high expression of SENP1 in TNBC tumors was significantly related to tumor lymph node metastasis and correlated with a short OS and RFS in TNBC patients. Using IHC, we showed that the expression of SENP1 was positively related to expression of CSN5. In further univariate and multivariate analyses of patients' clinicopathologic factors, we found that SENP1 expression was an independent factor for poor prognosis in TNBC. We further showed that by reducing SENP1 in TNBC subcutaneous mouse models and lung metastasis models, tumor growth and metastasis were significantly inhibited. Our results displayed an important role for SENP1 in TNBC progression and metastasis.

We used expression vectors that downregulate or overexpress SENP1 to study the role of SENP1 in TNBC cancer cell lines. Transwell migration/invasion and wound healing assays showed that up-regulation of SENP1 in TNBC cells could reduce their abilities in migration and invasion. We also checked expression of EMT markers, such as E-Cadherin, Krt12, Vimentin, Survivin, Slug, ZEB1 and ZEB2, in the TNBC cancer cells, and found that expression of ZEB1 was positively correlated with expression of SENP1. We also checked ZEB1 protein level at different time points to detect the half-life period of ZEB1, and observed that after SENP1 down-regulation, the half-life period of ZEB1 was reduced and degradation of ZEB1 was enhanced, indicating SENP1 regulates TNBC invasion and metastasis through regulation of ZEB1 degradation. ZEB1 modulates epithelial cell adhesion, and modulation of this adhesion is a key step for tumor cell invasion and metastasis 32,33. ZEB1 and ZEB2 are in one of the 3 families of transcription factors that promote EMT. Metastasis in many malignant tumors, such as pancreatic cancer, colon cancer and lung cancer, is related to expression of ZEB1 38-41. In many tumors, ZEB1 is regulated by the ubiquitination degradation pathway 42. Other studies showed that ZEB1 could promote tumor development via recruiting IL6 and IL8 in the tumor microenvironment 43. ZEB1 could inhibit miR-200 family, recruit SWI/SNF chromatin remodeling protein BRG1 and thus accelerate the EMT process 44,45. There are also studies found that ZEB1 is functioning in apical-basal polarity loss, which is important for the EMT and metastasis as well 46.

CSN5 regulates ubiquitin protein degradation pathways via deubiquitination of E3 ubiquitin ligase. CSN5 regulates CRL mediated ubiquitin protein degradation via deneddylation of E3 ubiquitin ligase, where the JAMM motif in CSN5 is the key structure for the deneddylation reaction 47. Our study displayed that ubiquitination of ZEB1 is significantly enhanced in shRNA-SENP1 breast cancer transfected group, compared with control group. According to bioinformatic data (http://ubibrowser.ncpsb.org/ubibrowser) as well as literatures 31,48, by using co-IP, we have tested the binding of ZEB1 with ubiquitinating enzymes (USP15, CSN5) and E3 ligases (MDM2, SMURF1, SYVN1), and found that ubiquitination of CSN5 regulates ZEB1 protein degradation. In a rescue experiment, we overexpressed CSN5 in SENP1 downregulated cells and found that CSN5 overexpression could reduce ZEB1 ubiquitination level and reverse the effects of SENP1 on EMT. According to ZEB1 Protein domain and amino acid arrangement, we predicted the ubiquitinating sites of ZEB1, and subsequently constructed expression vectors for ZEB1-K1108A, ZEB1-K186A, ZEB1-K439A. After transfection, we checked their ubiquitination level and found that CSN5 mainly mediates ubiquitination of ZEB1 at K1108, and thus regulates EMT in breast cancer.

GATA1 is an important transcription factor playing critical roles in hematopoiesis and tumor development 24-27. SUMO modification is an important form of protein modifications, and there are total four types of SUMO, namely, SUMO1, -2, -3 and -4. SUMOylation in GATA-1 is mainly marked by SUMO1. GATA-1 and C/EBPbeta are the two main transcription factors that bind to the promotor region of CSN5 29,49. Our ChIP and luciferase assay showed that reduced expression of SENP1 led to reduced protein level, mRNA level and promotor activity in CSN5. The binding of CSN5 promotor with GATA1 was reduced, while with C/EBPbeta was not changed. We further used co-IP to show that SENP1 affects GATA1 expression by regulating its SUMOylation level. This SUMOylation mediated effect was reversed in rescue experiment where GATA1-WT and GATA1-K137R expression vectors were transfected into SENP1 downregulated MDA-MB-231 cells. All these results indicated that SENP1 regulates CSN5 via regulation of GATA1 expression by modulating GATA1 lysine SUMOylation at K137.

Further, we have established subcutaneous tumor model and lung metastasis model, where we showed SENP1 downregulation could inhibit tumor development and metastasis into lung significantly. Immunohistochemistry analysis of these grown tumors showed that protein levels of ZEB1 and CSN5 were significantly reduced. We also used co-IP to detect GATA SUMOylation in tumors of different transplanted groups and validated that SENP1 regulates GATA1 SUMOylation. Meanwhile, high positive rate of CSN5, GATA1 and ZEB1 in cancer tissues detected by immunohistochemistry and we showed that expression of SENP1 is positively correlated with expression of CSN5, GATA1, ZEB1 in our TNBC samples.

As a summary, our study suggests that high expression of SENP1 in TNBC samples promotes TNBC tumor development and leads to poor prognosis. SENP1 regulates GATA1 SUMOylation, which further modulates CSN5 transcription. CNS5 attenuates ZEB1 ubiquitination which finally leads to altered ZEB1 expression, which is key for the EMT process in triple-negative breast cancer and leads to disease progression (Fig. 8). Several small-molecule inhibitors targeting SENP1 have been recently reported 50. Given the critical roles of SENP1 in TNBC invasion and metastasis, these compounds might be useful in the prevention and treatment of TNBC.

## Supplementary Material

Supplementary figures and tables.

## Author Contributions

All authors participated in designing the study. YCG performed the experiments and drafted the manuscript. RRW, JJL and XLQ analyzed the data. YCG, KZ performed the animal experiments. HL revised the manuscript for important intellectual content. XHH and YCG provided administrative, technical and material support. All authors read and approved the final manuscript.

## Figures and Tables

**Table 1:** Correlation of SENP1 expression to clinicopathologic features in TNBC.

Parameters	SENP1 -/+	SENP1 ++/+++	χ^2^	*P* value	r
Age (years)			2.399	0.121	0.127
<50	22	51			
≥50	31	42			
Tumor size			0.321	0.571	0.047
T1- T2	41	68			
T3- T4	12	25			
LN metastasis			9.564	0.023^a^	0.256
N0	23	25			
N1	21	30			
N2	6	21			
N3	3	17			
Histological grade			6.918	0.045^a^	0.202
G1	14	12			
G2	31	54			
G3	8	27			
Pathologic types			0.820	0.365	0.075
IDC	29	58			
Other	24	35			

Abbreviation: LN, lymph node; IDC, invasive ductal carcinoma ^a^Statistically significant (*P*< 0.05)

**Table 2:** Univariate and multivariate analysis of clinicopathologic factors for OS and RFS

Univariate analysis				
Variables	OS		RFS	
	HR (95.0% CI)	*P*	HR (95.0% CI)	*P*
Age(years)	0.899 (0.642-1.260)	0.538	1.082(0.771-1.519)	0.648
Tumor size	1.170(0.794-1.723)	0.428	1.287(0.875-1.893)	0.200
LN metastasis	1.663(1.403-1.970)	0.000^a^	1.548(1.300-1.843)	0.000^a^
Histological grade	1.656(1.268-2.161)	0.000^a^	1.427(1.097-1.857)	0.008^a^
Pathologic types	1.016(0.721-1.432)	0.928	0.934(0.663-1.315)	0.696
SENP1	2.771(1.882-4.079)	0.000^a^	2.319(1.471-3.110)	0.000^a^
**Multivariate analysis**				
LN metastasis	1.566(1.311-1.871)	0.000^a^	1.467(1.221-1.763)	0.001^a^
Histological grade	1.338(1.005-1.780)	0.046	1.149(0.870-1.517)	0.326
SENP1	2.552(1.724-3.778)	0.000^a^	1.933(1.317-2.836)	0.000^a^

Abbreviation: LN, lymph node; OS, overall survival; RFS, relapse-free survival aStatistically significant (P < 0.05)

## Abbreviation

SENP1: sentrin-specific protease 1
TNBC: Triple-negative breast cancer
GATA1: GATA binding protein 1
CSN5: COP9 signalosome complex subunit 5
ZEB1: Zinc finger E-box-binding homeobox 1
EMT: epithelial-mesenchymal transition
IHC: immunohistochemistry
TCGA: The Cancer Genome Atlas

## References

[1] DeSantis CE, Ma J, Gaudet MM, Newman LA, Miller KD, Goding Sauer A (2019). Breast cancer statistics,2019. CA Cancer J Clin.

[2] Siegel RL, Miller KD, Jemal A (2020). Cancer statistics, 2020. CA Cancer J Clin.

[3] Tomao F, Papa A, Zaccarelli E, Rossi L, Caruso D, Minozzi M (2015). Triple-negative breast cancer: new perspectives for targeted therapies. Onco Targets Ther.

[4] Haffty BG, Yang Q, Reiss M, Kearney T, Higgins SA, Weidhaas J (2006). Locoregional relapse and distant metastasis in conservatively managed triple negative early-stage breast cancer. J Clin Oncol.

[5] Elias AD (2010). Triple-negative breast cancer: a short review. Am J Clin Oncol.

[6] Anders CK, Deal AM, Miller CR, Khorram C, Meng H, Burrows E (2011). The prognostic contribution of clinical breast cancer subtype, age, and race among patients with breast cancer brain metastases. Cancer.

[7] Niwifiska A, Murawska M, Pagoda K (2010). Breast cancer brainmetastases: differences in survival depending on biological subtype, RPA RTOG prognostic class and systemic treatment after whale-brain radiotherapy(WBRT). Ann Oncol.

[8] Dent R, Trudeau M, Pritchard KI, Hanna WM, Kahn HK, Sawka CA (2007). Triple-negative breast cancer: clinical features and patterns of recurrence. Clin Cancer Res.

[9] Perou CM, Sørlie T, Eisen MB, van de Rijn M, Jeffrey SS, Rees CA (2000). Molecular portraits of human breast tumours. Nature.

[10] Sørlie T, Wang Y, Xiao C, Johnsen H, Naume B, Samaha RR (2006). Distinct molecular mechanisms underlying clinically relevant subtypes of breast cancer: gene expression analyses across three different platforms. BMC Genomics.

[11] Saitoh H, Hinchey J (2000). Functional heterogeneity of small ubiquitin-related proteinmodifiers SUMO-1 versus SUMO-2/3. J Biol Chem.

[12] Melchior F (2000). SUMO-nonclassical ubiquitin. Annu Rev Cell Dev Biol.

[13] Hay RT (2007). SUMO-specific proteases: a twist in the tail. Trends Cell Biol.

[14] Hickey CM, Wilson NR, Hochstrasser M (2012). Function and regulation of SUMO proteases. Nat Rev Mol Cell Biol.

[15] Mukhopadhyay D, Dasso M (2007). Modification in reverse: the SUMO proteases. Trends Biochem Sci.

[17] Bawa-Khalfe T, Yeh ET (2010). SUMO losing balance: SUMO proteases disrupt SUMO homeostasis to facilitate cancer development and progression. Genes Cancer.

[18] Bettermann K, Benesch M, Weis S, Haybaeck J (2012). SUMOylation in carcinogenesis. Cancer Lett.

[19] Gong L, Millas S, Maul GG, Yeh ET (2000). Differential regulation of sentrinized proteins by a novel sentrin-specific protease. J Biol Chem.

[20] Cheng J, Bawa T, Lee P, Gong L, Yeh ET (2006). Role of desumoylation in the development of prostate cancer. Neoplasia.

[21] Wang Q, Xia N, Li T, Xu Y, Zou Y, Zuo Y (2013). SUMO-specific protease 1 promotes prostate cancer progression and metastasis. Oncogene.

[22] Wang Z, Jin J, Zhang J, Wang L, Cao J (2016). Depletion of SENP1 suppresses the proliferation and invasion of triple-negative breast cancer cells. Oncol Rep.

[23] Wilkinson-White L, Lester KL, Ripin N, Jacques DA, Mitchell Guss J, Matthews JM (2015). GATA1 directly mediates interactions with closely spaced pseudopalindromic but not distantly spaced double GATA sites on DNA. Protein Sci.

[24] Gilles L, Arslan AD, Marinaccio C, Wen QJ, Arya P, McNulty M (2017). Downregulation of GATA1 drives impaired hematopoiesis in primary myelofibrosis. J Clin Invest.

[25] Li Y, Ke Q, Shao Y, Zhu G, Li Y, Geng N (2015). GATA1 induces epithelialmesenchymal transition in breast cancer cells through PAK5 oncogenic signaling. Oncotarget.

[26] Boidot R, Végran F, Jacob D, Chevrier S, Cadouot M, Feron O (2010). The transcription factor GATA-1 is overexpressed in breast carcinomas and contributes to survivin upregulation via a promoter polymorphism. Oncogene.

[27] Tsang AP, Visvader JE, Turner CA, Fujiwara Y, Yu C, Weiss MJ (1997). FOG, a multitype zinc finger protein, acts as a cofactor for transcription factor GATA-1 in erythroid and megakaryocytic differentiation. Cell.

[28] Yu L, Ji W, Zhang H, Renda MJ, He Y, Lin S (2010). SENP1-mediated GATA1 deSUMOylation is critical for definitive erythropoiesis. J Exp Med.

[29] Shackleford TJ, Zhang Q, Tian L, Vu TT, Korapati AL, Baumgartner AM (2011). Stat3 and CCAAT/enhancer binding protein beta (C/EBP-beta) regulate Jab1/CSN5 expression in mammary carcinoma cells. Breast Cancer Res.

[30] Wang S, Oh DY, Leventaki V, Drakos E, Zhang R, Sahin AA (2019). MicroRNA-17 acts as a tumor chemosensitizer by targeting JAB1/CSN5 in triple-negative breast cancer. Cancer Lett.

[31] Zhang S, Hong Z, Chai Y, Liu Z, Du Y, Li Q (2017). CSN5 promotes renal cell carcinoma metastasis and EMT by inhibiting ZEB1 degradation. Biochem Biophys Res Commun.

[32] Hou P, Li L, Chen F, Chen Y, Liu H, Li J (2018). PTBP3-mediated regulation of ZEB1 mRNA stability promotes epithelial-mesenchymal transition in breast cancer. Cancer Res.

[33] Liang W, Song S, Xu Y, Li H, Liu H (2018). Knockdown of ZEB1 suppressed the formation of vasculogenic mimicry and epithelial-mesenchymal transition in the human breast cancer cell line MDA- MB- 231. Mol Med Rep.

[34] Vivian J, Rao AA, Nothaft FA, Ketchum C, Armstrong J, Novak A (2017). Toil enables reproducible, open source, big biomedical data analyses. Nat biotechnol.

[35] Xiang-Ming Y, Zhi-Qiang X, Ting Z, Jian W, Jian P, Li-Qun Y (2016). SENP1 regulates cell migration and invasion in neuroblastoma. Biotechnol Appl Biochem.

[36] Sun XX, Chen Y, Su Y, Wang X, Chauhan KM, Liang J (2018). SUMO protease SENP1 deSUMOylates and stabilizes c-Myc. Proc Natl Acad Sci U S A.

[37] Huang X, Zuo Y, Wang X, Wu X, Tan H, Fan Q (2019). SUMO-Specific Protease 1 Is critical for myeloid-derived suppressor cell development and function. Cancer Res.

[38] Gheldof A, Hulpiau P, van Roy F, De Craene B, Berx G (2012). Evolutionary functional analysis and molecular regulation of the ZEB transcription factors. Cell Mol Life Scie.

[39] Guo X, Zhao L, Cheng D, Mu Q, Kuang H, Feng K (2017). AKIP1 promoted epithelial-mesenchymal transition of non-small-cell lung cancer via transactivating ZEB1. Am J Cancer Res.

[40] Larsen JE, Nathan V, Osborne JK, Farrow RK, Deb D, Sullivan JP (2016). ZEB1 drives epithelial-to-mesenchymal transition in lung cancer. J Clin Invest.

[41] Zhang M, Miao F, Huang R, Liu W, Zhao Y, Jiao T (2018). RHBDD1 promotes colorectal cancer metastasis through the Wnt signaling pathway and its downstream target ZEB1. J Exp Clin Cancer Res.

[42] Inoue Y, Itoh Y, Sato K, Kawasaki F, Sumita C, Tanaka T (2016). Regulation of epithelial-mesenchymal transition by E3 ubiquitin ligases and deubiquitinase in cancer. Curr Cancer Drug Targets.

[43] Katsura A, Tamura Y, Hokari S, Harada M, Morikawa M, Sakurai T (2017). ZEB1-regulated inflammatory phenotype in breast cancer cells. Mol Oncol.

[44] Sánchez-Tilló E, Lázaro A, Torrent R, Cuatrecasas M, Vaquero EC, Castells A (2010). ZEB1 represses E-cadherin and induces an EMT by recruiting the SWI/SNF chromatin-remodeling protein BRG1. Oncogene.

[45] Díaz-López A, Díaz-Martín J, Moreno-Bueno G, Cuevas EP, Santos V, Olmeda D (2015). Zeb1 and Snail1 engage miR-200f transcriptional and epigenetic regulation during EMT. Int J Cancer.

[46] Spaderna S, Schmalhofer O, Wahlbuhl M, Dimmler A, Bauer K, Sultan A (2008). The transcriptional repressor ZEB1 promotes metastasis and loss of cell polarity in cancer. Cancer Res.

[47] Echalier A, Pan Y, Birol M, Tavernier N, Pintard L, Hoh F (2013). Insights into the regulation of the human COP9 signalosome catalytic subunit, CSN5/Jab1. Proc Natl Acad Sci USA.

[48] Zhou Z, Zhang P, Hu X, Kim J, Yao F, Xiao Z (2017). USP51 promotes deubiquitination and stabilization of ZEB1. Am J Cancer Res.

[49] Collavin L, Gostissa M, Avolio F, Secco P, Ronchi A, Santoro C (2004). Modification of the erythroid transcription factor GATA-1 by SUMO-1. Proc Natl Acad Sci USA.

[50] Jia Y, Claessens LA, Vertegaal ACO, Ovaa H (2019). Chemical tools and biochemical assays for SUMO specific proteases(SENPs). ACS Chem Biol.

