# Supplementary material for: SENP1 promotes triple-negative breast cancer invasion and metastasis via enhancing CSN5 transcription mediated by GATA1 deSUMOylation
Source: Int J Biol Sci. 2022 Mar 6;18(5):2186–201. doi: 10.7150/ijbs.60594 (PMC8935229; doi:10.7150/ijbs.60594)
Supplement: Supplementary file 1 — Supplementary figures and tables. [file ijbsv18p2186s1.pdf]

Supplementary Figure 1

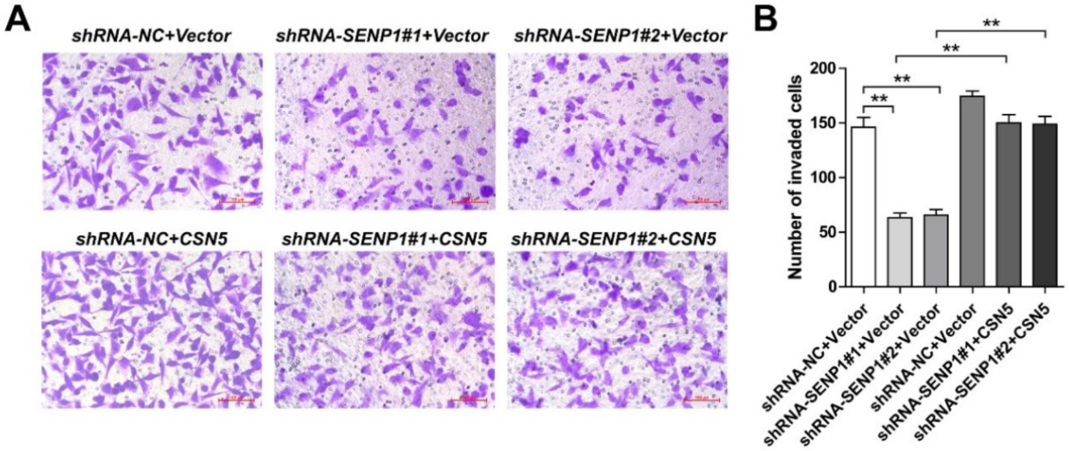

Supplementary Figure 1. SENP1 affects cell invasion through CSN5. The MDA-MB-231 cells were transfected with shRNA-NC+Vector, shRNA-SENP1#1+Vector, shRNASENP1#2+Vector, shRNA-NC+Vector, shRNA-SENP1#1+CSN5 or shRNASENP1#2+CSN5. MDA-MB-231 cells were seeded on chambers. Cells that migrated through the matrigel-coated chambers (A, B) were stained with crystal violet. Results were mean ± SD for three individual experiments. **P*<0.05, ***P*<0.01

Supplementary Figure 2

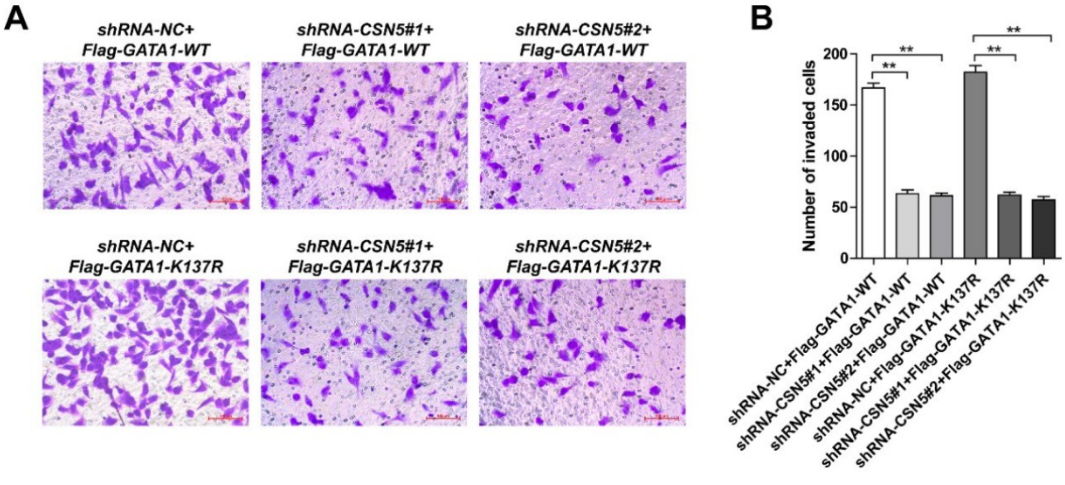

Supplementary Figure 2. GATA1 affects the invasive ability of MDA-MB-231 by regulating CSN5. The MDA-MB-231 cells were transfected with shRNA-NC+Flag-GATA1-WT,shRNA-CSN5#1+Flag-GATA1-WT, shRNA-CSN5#2+Flag-GATA1-WT, shRNANC+Flag-GATA1-K137R, shRNA-CSN5#1+Flag-GATA1-K137R or shRNACSN5#2+Flag-GATA1-K137R. MDA-MB-231 cells were seeded on

chambers. Cells that migrated through the matrigel-coated chambers (A, B) were stained with crystal violet. Results were mean ± SD for three individual experiments. *P<0.05, **P<0.01.

Supplementary table S1. **Primers used in Quantitative Real-Time PCR.**

Primers	Sequence (5’→3’)	
SENP1	Forward	CAGTGATGTGAAAAGACTTGA
	Reverse	GTCCTTGCCTGGAAGATAAA
ZEB1	Forward	AGATGTTTCCTTCCAATCCA
	Reverse	CAATAGCCTATGCTCCACTC
CSN5	Forward	GCATATGAATACATGGCTGC
	Reverse	GCTGATTGAGCATCTGAGTA
GAPDH	Forward	ATGAATGGGCAGCCGTTA
	Reverse	CAATACGACCAAATCAGAGAAT

Supplementary table S2. Antibodies and dilution(Western blotting analysis).

Antibodies	Dilution (application)
SENP1	1:1000 (WB)
	1:500 (IHC)
E-cadherin	1:10000 (WB)
Krt12	1:5000 (WB)
vimentin	1:1000 (WB)

survivin	1:5000 (WB)
Snail	1:1000 (WB)
Slug	1 μg/ml
twist	1:1000 (WB)
ZEB1	1:1000 (WB)<br>1:500 (IP)
ZEB2	1:1000 (WB)
CSN5	1:1000 (WB)<br>5 μg/ml (IHC)
GATA1	1:10000 (WB)<br>1:100 (CHIP)
USP51	1:1000 (WB)
MDM2	1:1000 (WB)
SMURF1	1 μg/ml (WB)
SYVN1	1:2000 (WB)
SUMO1	1:1000 (WB)
GAPDH	1:1000 (WB)
Flag	1:50 (IP)<br>1:1000 (WB)
C/EBPbeta	1:20 (CHIP)

Goat Anti-Rabbit IgG H&L(HRP)	1:10000 (WB)
Rabbit Anti-Mouse IgG H&L (HRP)	1:10000 (WB)

Supplementary table S3. Primers used **in plasmid constructs, gene expression alteration and determination.**

		Sequence (5'→3')
CSN5	Forward	5'-CTAGCGGCCGCTGGTTGTAGAGACGACAACTT-3'
	Reverse	5'-ATTTTCTAGAAAGTACTTCTCAGAGACTGTT-3'
GATA1	Forward	5'-CTAGCGGCGAGTTCCCTGGCCTGGGGTCC-3'
	Reverse	5'-ATTTTCTAGA TCATGAGCTGAGCGGAGCCAC-3'
ZEB1	Forward	5'-CTAGCGGCCGC ATGGCGGATGGCCC-3'
	Reverse	5'-ATTTTCTAGATTAGGCTTCATTTGTCTTTTCTTCA-3'
GATA1-K137R	Forward	5'-CTTTGCGGACAGAGCGGCTGAGC-3'
	Reverse	5'- CTGTCCGCAAAGTCTCCAGGAAGCTGG-3'
ZEB1-K1108A	Forward	5'- CAAGCTGTAGGCGAGAGTAGTGAG-3'
	Reverse	5'- GCCTACAGCTTGTCCTAAGCTGCTTGCTT-3'
ZEB1-K186A	Forward	5'-CTGGCTGAACACATTAAATATCGTCATGAAAAGA-3'
	Reverse	5'-GTGTTCAGCCAGAGAGGTAAAGCGTTTATAGC-3'
ZEB1-K439A	Forward	5'-CATCCGCTGAACAAGAAACAATCAATGCTTCACCC-3'
	Reverse	5'-GTTCAGCGGATGCAAGATTGGCTTGATTATTC-3'
